# Predictive Models Using Machine Learning to Identify Fetal Growth Restriction in Patients With Preeclampsia: Development and Evaluation Study

**DOI:** 10.2196/70068

**Published:** 2025-05-27

**Authors:** Qing Hua, Fengchun Yang, Yadan Zhou, Fenglian Shi, Xiaoyan You, Jing Guo, Li Li

**Affiliations:** 1 Department of Obstetrics and Gynecology Zhengzhou Central Hospital Affiliated to Zhengzhou University Zhengzhou China; 2 Institute of Medical Information Chinese Academy of Medical Sciences & Peking Union Medical College Beijing China; 3 Department of Infectious Diseases Tongji Hospital Tongji Medical College and State Key Laboratory for Diagnosis and Treatment of Severe Zoonostic Infectious Disease, Huazhong University of Science and Technology Wuhan China

**Keywords:** machine learning, fetal growth restriction, preeclampsia, random forest, Shapley additive explanations

## Abstract

**Background:**

Fetal growth restriction (FGR) is a common complication of preeclampsia. FGR in patients with preeclampsia increases the risk of neonatal-perinatal mortality and morbidity. However, previous prediction methods for FGR are class-biased or clinically unexplainable, which makes it difficult to apply to clinical practice, leading to a relative delay in intervention and a lack of effective treatments.

**Objective:**

The study aims to develop an auxiliary diagnostic model based on machine learning (ML) to predict the occurrence of FGR in patients with preeclampsia.

**Methods:**

This study used a retrospective case-control approach to analyze 38 features, including the basic medical history and peripheral blood laboratory test results of pregnant patients with preeclampsia, either complicated or not complicated by FGR. ML models were constructed to evaluate the predictive value of maternal parameter changes on preeclampsia combined with FGR. Multiple algorithms were tested, including logistic regression, light gradient boosting, random forest (RF), extreme gradient boosting, multilayer perceptron, naive Bayes, and support vector machine. The model performance was identified by the area under the curve (AUC) and other evaluation indexes. The Shapley additive explanations (SHAP) method was adopted to rank the feature importance and explain the final model for clinical application.

**Results:**

The RF model performed best in discriminative ability among the 7 ML models. After reducing features according to importance rank, an explainable final RF model was established with 9 features, including urinary protein quantification, gestational week of delivery, umbilical artery systolic-to-diastolic ratio, amniotic fluid index, triglyceride, D-dimer, weight, height, and maximum systolic pressure. The model could accurately predict FGR for 513 patients with preeclampsia (149 with FGR and 364 without FGR) in the training and testing dataset (AUC 0.83, SD 0.03) using 5-fold cross-validation, which was closely validated for 103 patients with preeclampsia (n=45 with FGR and n=58 without FGR) in an external dataset (AUC 0.82, SD 0.048). On the whole, urinary protein quantification, umbilical artery systolic-to-diastolic ratio, and gestational week of delivery exhibited the highest contributions to the model performance (*c*=0.45, 0.34, and 0.33) based on SHAP analysis. For specific individual patients, SHAP results reveal the protective and risk factors to develop FGR for interpreting the model’s clinical significance. Finally, the model has been translated into a convenient web page tool to facilitate its use in clinical settings.

**Conclusions:**

The study successfully developed a model that accurately predicts FGR development in patients with preeclampsia. The SHAP method captures highly relevant risk factors for model interpretation, alleviating concerns about the “black box” problem of ML techniques.

## Introduction

Preeclampsia is a hypertensive disorder of pregnancy that originates at the maternal-fetal interface and affects multiple organ systems with an incidence rate ranging from 2% to 8% [1,2]. Severe forms of preeclampsia can lead to complications such as maternal death, fetal growth restriction (FGR), and stillbirth [3]. The research data from China revealed that the incidence rate of FGR in early-onset preeclampsia is as high as 59.1% [4].

FGR is defined as the failure of a fetus to achieve its genetic growth potential [5]. They have an increased risk of perinatal or long-term complications, such as fetal asphyxia, neurodevelopmental disabilities, cardiovascular disease, and type 2 diabetes, compared with normally grown fetuses [6,7]. It is often accompanied by preeclampsia, which may result in increased perinatal mortality. Thus the early identification and intervention of FGR are crucial for improving its perinatal outcomes. However, early prediction methods are limited, and even in developed countries, more than 50% of FGR cases are not detected before birth [8], leading to a relative delay in intervention and a lack of effective treatments.

Ultrasound measurements have been widely validated for identifying FGR, yet there is still a certain rate of missed diagnosis [9]. This is because ultrasound measurements serve as bone markers, ignoring soft tissues like fetal fat and muscle, which can lead to estimation errors [10]. Besides, errors can also arise from the selection of section surfaces and the influence of fetal position [11]. If a feasible prediction method can be identified, it would be highly valuable in improving outcomes for preeclampsia complicated by FGR.

An appropriate prediction model is needed for patients with preeclampsia accompanied by FGR. Several studies have attempted to develop models for the prediction of FGR. For example, Feng et al [12] established a combined first- and second-trimester prediction model for screening late-onset FGR in fetuses using multivariate logistic regression. Machine learning (ML) algorithms are increasingly favored for such tasks due to their superior ability to handle complex, nonlinear relationships [13]. For instance, Huang et al [14] developed an artificial neural network model to predict the occurrence of preeclampsia complicated by FGR based on maternal peripheral blood parameters and clinical indicators. However, most existing models were always developed from datasets with highly imbalanced classes [15]. Imbalanced data causes model bias, overfitting, feature importance distortion, and unreliable performance metrics due to the dominance of the majority class. This issue is particularly pronounced in preeclampsia and FGR prediction, where class imbalance can lead to overly optimistic model performance estimates [16]. In addition, previous studies tend to focus narrowly on specific subgroups, such as early- or late-onset FGR or preeclampsia [17], limiting their generalizability. Furthermore, the inherent “black box” of ML models prevents clear interpretation of predictive features, which poses a barrier to clinical adoption. In summary, existing prediction models suffer from narrow population scope, class imbalance, and lack of clinical interpretability.

Considering that FGR often coexists with preeclampsia, it is of great significance to predict the occurrence of FGR in patients with preeclampsia. Thus, the study aims to develop an auxiliary diagnostic model based on ML to predict the occurrence of FGR in patients with preeclampsia. Specifically, we constructed and optimized the ML model for predicting the occurrence of FGR in patients with preeclampsia. Meanwhile, in order to facilitate clinical understanding and application, we adopted the Shapley additive explanations (SHAP) method to capture highly relevant risk factors for model interpretation.

## Methods

### Data Collection

We selected patients with preeclampsia who gave birth in the Obstetrics Department of Zhengzhou Central Hospital Affiliated to Zhengzhou University from January 2021 to April 2024. The gestational age was determined based on the fetus size indicated by the last menstrual period and early pregnancy color ultrasound. If there was inconsistency between the two methods, the determination was made based on the results of the early pregnancy color ultrasound.

The diagnosis of preeclampsia is based on the American College of Obstetricians and Gynecologists criteria [2]: (1) systolic blood pressure ≥140 mm Hg, diastolic blood pressure ≥90 mm Hg after 20 weeks of gestation, or both, accompanied by urine protein quantification of 300 mg/24 hours, or random urine protein (+); (2) in the absence of proteinuria, preeclampsia can also be diagnosed if newly diagnosed hypertension occurs along with thrombocytopenia (platelet count <100×10^9^/L), impaired liver function (transaminase 2 times higher than the normal upper limit), new development of renal insufficiency (serum creatinine >97 umol/L or higher than 2 times the normal upper limit), pulmonary edema, or new onset cerebral or visual disturbances. FGR diagnostic criteria (Chinese population’s birth weight chart) [7] are as follows: the estimated fetal weight below the 10th percentile compared with the population norms on growth charts. Specifically, fetuses whose birth weight is less than 10% of the standard growth value for the corresponding gestational week are defined as FGR, and the others are non-FGR.

**Figure 1:**
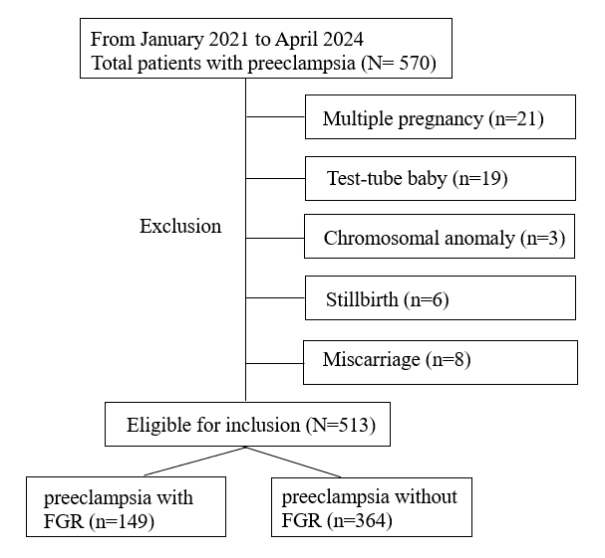
Flowchart of patient selection. FGR: fetal growth restriction.

After excluding patients with multiple pregnancies, test-tube babies, fetal chromosomal anomalies, miscarriage in the second trimester, stillbirth, and incomplete medical records, we included a total of 513 cases for analysis among 570 patients with preeclampsia, consisting of 149 patients with FGR and 364 patients without FGR. The selection of research objects is shown in Figure 1. Following the same criteria as mentioned earlier, we also collected a dataset for external validation of the model. This dataset includes records from 103 pregnant women, among whom 45 delivered infants with FGR and 58 without FGR.

We initially identified 38 features potentially related to FGR through a literature review. Recorded patient characteristics included baseline characteristics, laboratory results in late pregnancy (before delivery), and the weight of neonates. Baseline characteristics included maternal age, gravidity, parity, prepregnancy BMI, blood pressures, gestational ages, personal history (preeclampsia, repeated abortions, and missed abortion history), family history (hypertensive disorders), with or without chronic hypertension, coexistence of gestational diabetes mellitus during pregnancy, systolic-to-diastolic ratio (S/D) value, amniotic fluid index (AFI), and gestational age at birth. Relevant laboratory tests included urine protein quantitation, routine blood tests, hepatic and renal function, lipid profiles, coagulation function, and serum haptoglobin. In past studies, these features of preeclampsia were strongly associated with FGR. Subsequently, we used feature importance to select more critical features for the final model construction.

### Ethical Considerations

The protocol was approved by the ethics committee of Zhengzhou Central Hospital Affiliated to Zhengzhou University (no. ZXYY202470). Considering its retrospective nature, the requirement for informed consent was waived. All relevant clinical data and outcome information were collected from the hospital’s electronic medical record system.

### Model Training and Validation

Before model training, we applied identical preprocessing steps to the training, validation, and test sets. First, we removed variables with missing rates exceeding 10%, then imputed the remaining missing values using a random forest (RF)–based method, missForest. For continuous variables, we conducted standardization through min-max scaling. We divided the collected maternal data into a training set and a test set, with 70% used for training and 30% for validation (internal validation), in order to avoid problems with overﬁtting. The SHAP method was adopted to rank the feature importance. For feature selection, we added features to the model training process step by step according to the order of feature importance shown by SHAP.

For training models, we applied 7 widely used ML algorithms in assisted diagnosis prediction, including logistic regression [18], tree-based model (light gradient boosting machine [LightGBM]) [19], RF [20], and extreme gradient boosting (XGBoost) [21], multilayer perceptron [22], naive Bayes [23], and support vector machine–based methods [24]. The logistic regression model represents the linear model, which is a state-of-the-art classification model for baseline construction. RF, LightGBM, and XGBoost were representative ensemble learning models, which do well in dealing with multiple types of features. RF is a bagging ensemble algorithm containing multiple decision trees, which uses the voting method to classify samples and integrate the final voting results produced by multiple decision trees. XGBoost is an optimized distributed gradient boosting library designed to be highly efficient, flexible, and portable. It implements ML algorithms under the gradient boosting framework. LightGBM is a gradient-boosting framework that uses tree-based learning algorithms. It uses a gradient-based 1-sided sampling algorithm to reduce the sample dimension and a mutually exclusive feature bundling algorithm to reduce the feature dimension.

A total of 38 features aforementioned were used to develop the prediction models. In order to optimize the prediction model, grid search combined with manual ﬁne-tuning was applied to obtain the ﬁnal hyperparameters (Multimedia Appendix 1). Commonly used evaluation indexes, such as the area under the receiver operating characteristic curve (AUC-ROC), sensitivity (recall), speciﬁcity, accuracy, precision, and *F*_1_-score were used to evaluate the reliability of these models. During the training of our prediction models, we used the 5-fold cross-validation method for model training. The SHAP method was able to select clinically important features that contribute significantly to model predictions. It can improve the robustness of the model to a certain extent by removing irrelevant or noisy features and avoiding the feature collinearity problem. Finally, the prediction model was further evaluated in an external dataset.

### Model Interpretation

To enhance the clinical applicability, we combined the diagnostic prediction results with clinical feature importance explanations to identify a patient with preeclampsia and FGR. In this study, except for interpreting feature contributions, SHAP values were used to assess the clinical significance of predictive models [25].

For the ML model interpretation, we used both globally explainable and locally explainable methods [26]. The global explanation determines the importance of features by comparing the magnitude of the model prediction error change before and after replacing a feature. If the prediction error changes more, it indicates that the feature is more important. Meanwhile, we used SHAP values to explore individual-based decision-making processes in the view of local explanation.

### Model Deployment

Web page deployment tool based on the Streamlit framework to facilitate the use of the model in clinical settings, the ﬁnal prediction model was implemented into a web application established based on the Streamlit Python-based framework (Python Software Foundation). When the values of corresponding features from the ﬁnal model are provided, the application can return the probability of FGR and the force plot for the individual.

## Results

### Patient Characteristics

We collected information on 513 parturients and newborns, of which 149 were diagnosed with FGR. Among the information collected before delivery, 29 variables were continuous and 9 were discrete. We statistically analyzed information on the group differences between these variables between newborns with FGR and those without FGR. Demographics, pregnancy characteristics, and neonatal outcomes for eligible patients with preeclampsia are described in Table 1. Maternal demographics in regard to age, height, and prepregnancy BMI have no difference between the 2 groups (*P*<.05) but differed in gestational age at diagnosis of preeclampsia, weight, weight gain during pregnancy, maximum systolic or diastolic pressure (mm Hg), umbilical artery (S/D), and AFI. Previous preeclampsia, onset period of hypertension, chronic hypertension, combined with gestational diabetes, history of FGR, and family history of hypertension were strong risk factors for FGR in patients with preeclampsia in our cohort. The following laboratory parameters with statistically significant differences were screened by intergroup difference analysis (*P*<.05): hemoglobin, total protein, albumin, uric acid, fibrinogen, total cholesterol, and serum haptoglobin (Table 1). Besides, there was also a difference in the sex of the newborns between the 2 groups.

**Table 1:** Comparison of demographic and clinical characteristics and outcomes between non-FGR^a^ and FGR in the cohort.

	Non-FGR group (n=364)	FGR group (n=149)	*P* value
Age (years), mean (SD)	31.24 (4.43)	31.81 (4.55)	.29
Onset period of hypertension (weeks), mean (SD)	32.3 (10.68)	30.91 (9.73)	.19
Gestational age at diagnosis of preeclampsia (weeks), mean (SD)	36.73 (3.71)	34.48 (4.49)	<.001
Gestational week of delivery (weeks), mean (SD)	37.81 (2.32)	35.45 (3.6)	<.001
Maximum systolic pressure (mm Hg), mean (SD)	152.95 (15.92)	173.56 (146.12)	.01
Maximum diastolic pressure (mm Hg), mean (SD)	100.44 (10.64)	111.79 (87.52)	.02
Height (cm), mean (SD)	161.28 (4.75)	160.41 (4.65)	.07
Weight (kg), mean (SD)	78.8 (11.55)	74.98 (10.91)	<.001
Prepregnancy BMI (kg/m^2^), mean (SD)	24.54 (4.17)	23.89 (3.86)	.12
Weight gain during pregnancy (kg), mean (SD)	15.05 (7.59)	13.28 (4.64)	.01
Urinary protein quantification (mg/24 h), mean (SD)	1362.1 (2310.7)	3032.6 (3678.96)	<.001
Platelets (×10^9^/L), mean (SD)	197.05 (65.93)	188.67 (60.65)	.20
Hematocrit (%), mean (SD)	36.1 (20.5)	36.4 (4.2)	.88
Hemoglobin (g/L), mean (SD)	116.33 (15.84)	121.63 (14.91)	<.001
Total protein (g/L), mean (SD)	58.75 (6.11)	57.15 (7.19)	.01
Albumin (g/L), mean (SD)	31.79 (4.38)	30.9 (3.75)	.04
Globulin (g/L), mean (SD)	26.97 (3.83)	26.45 (3.97)	.12
Alanine transferase (U/L), mean (SD)	22.64 (47.85)	26.44 (35.88)	.40
Aspartate transaminase (U/L), mean (SD)	31.5 (24.74)	34.21 (32.11)	.32
Alkaline phosphatase (U/L), mean (SD)	163.68 (85.42)	150.33 (63.63)	.10
Total bilirubin (umol/L), mean (SD)	11.83 (6.73)	13.44 (24.19)	.27
Uric acid (umol/L), mean (SD)	369.88 (101.3)	405.6 (107.17)	<.001
D-dimer (mg/L), mean (SD)	2.1 (1.71)	2.12 (3.71)	.95
Fibrinogen (mg/L), mean (SD)	4.33 (1.07)	4.07 (1.1)	.02
Total cholesterol (mmol/L), mean (SD)	6.48 (1.7)	8.32 (15.34)	.03
Triglyceride (mmol/L), mean (SD)	4.74 (2.57)	4.62 (2.79)	.64
Serum haptoglobin (g/L), mean (SD)	0.7 (0.35)	0.57 (0.35)	<.001
Amniotic fluid index (mm), mean (SD)	115.5 (36.75)	103.45 (32.6)	<.001
Umbilical artery S/D^b^ (free segment), mean (SD)	2.33 (0.74)	2.73 (0.85)	<.001
Chronic hypertension (hypertension diagnosed before 20 weeks of pregnancy), n (%)	428 (83.4)	85 (16.6)	.76
History of FGR, n (%)	500 (97.4)	13 (2.5)	<.001
History of cesarean section, n (%)	384 (74.8)	129 (25.2)	.83
History of adverse pregnancy, n (%)	416 (81.1)	97 (18.9)	.07
Primipara, n (%)	219 (42.6)	294 (57.3)	.99
Combined with gestational diabetes, n (%)	365 (71.2)	148 (28.8)	.60
Family history of hypertension, n (%)	424 (82.6)	89 (17.3)	.93
Fetal sex, n (%)	243 (47.3)	270 (52.6)	<.001

^a^FGR: fetal growth restriction.

^b^S/D: systolic-to-diastolic ratio.

### Model Development and Performance Comparison

**Figure 2:**
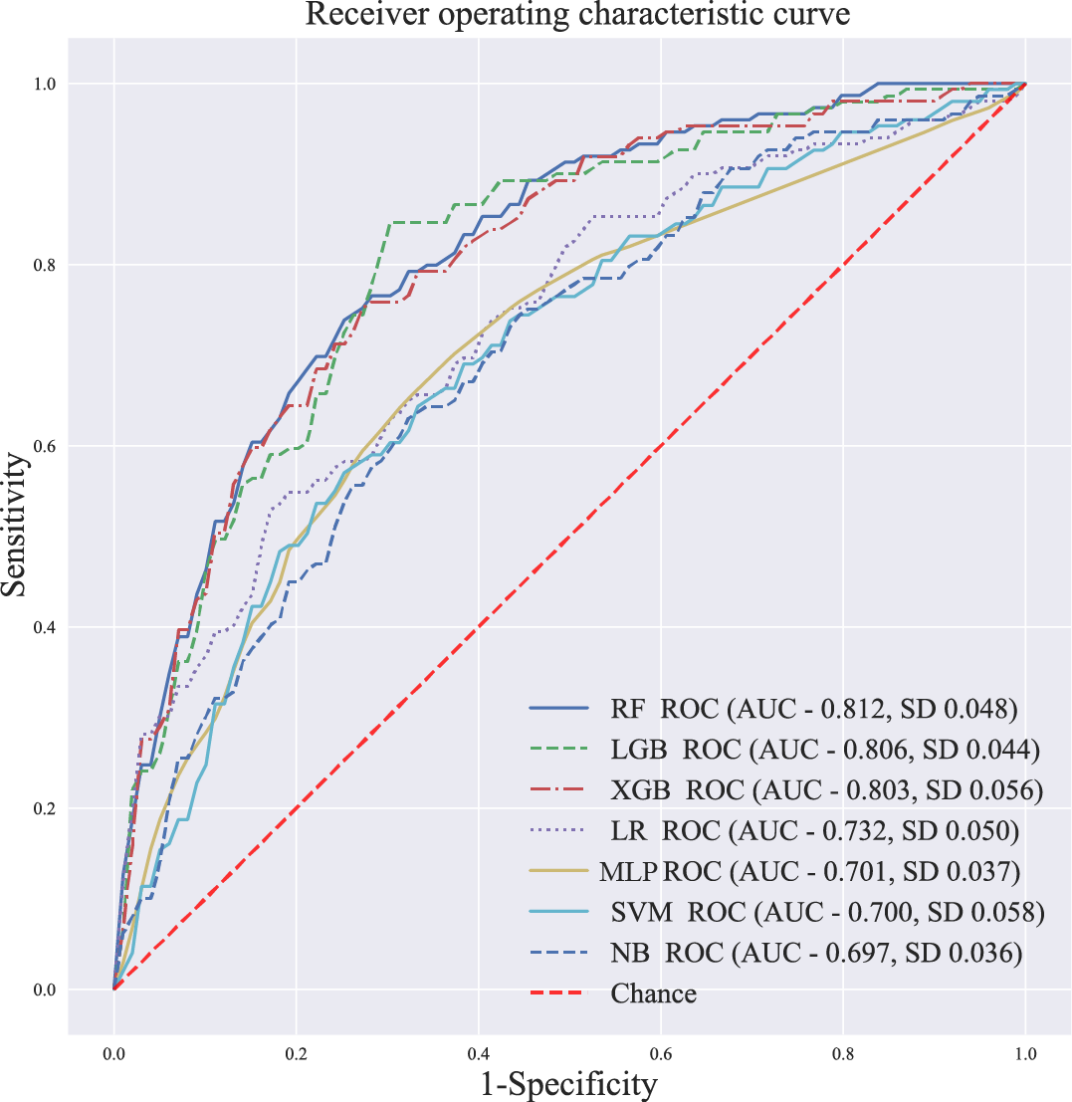
ROC curves of the 7 machine learning models. AUC: area under the curve; LGB: light gradient boosting; LR: logistic regression; MLP: multilayer perceptron; NB: naive Bayes; RF: random forest; ROC: receiver operating characteristic; SVM: support vector machine; XGB: extreme gradient boosting.

**Figure 3:**
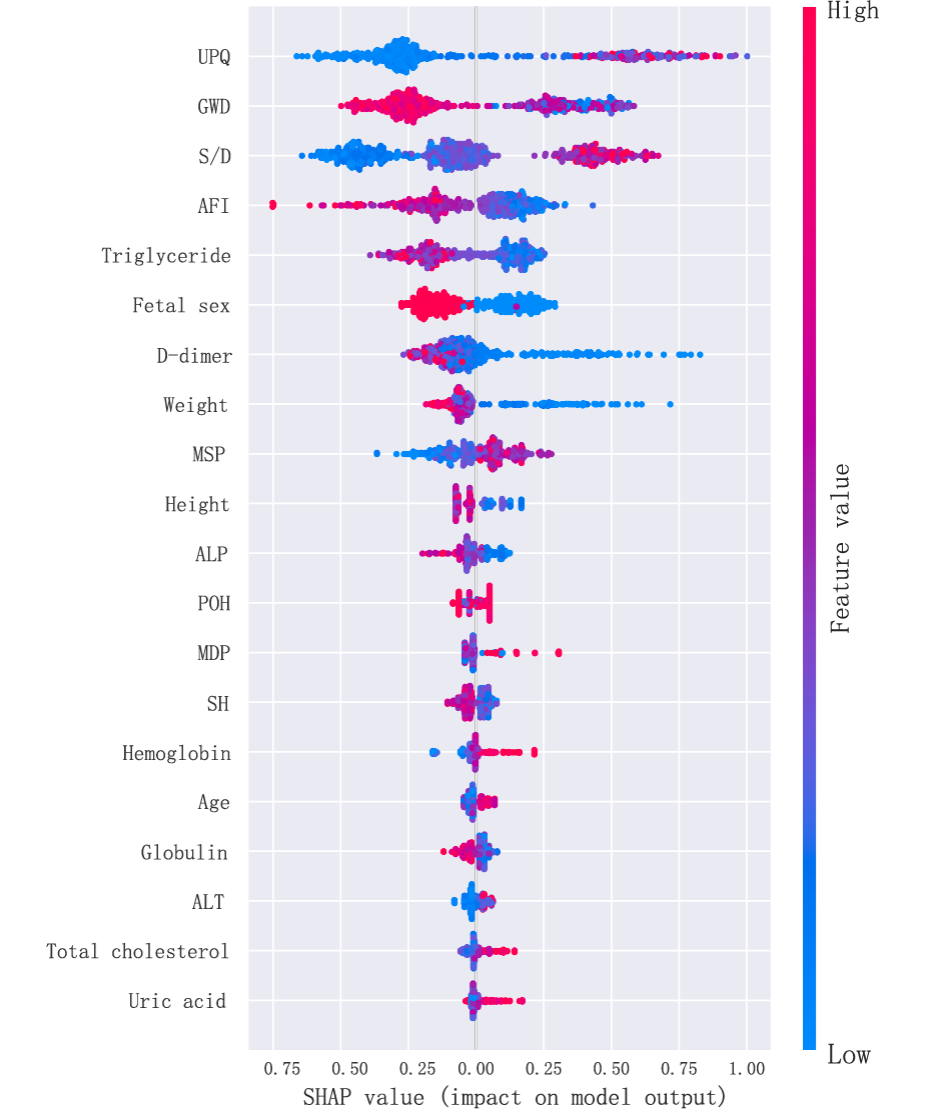
The SHAP summary plots of the top 20 features for the RF model. AFI: amniotic fluid index; ALP: alkaline phosphatase; GWD: gestational week of delivery; MDP: maximum diastolic pressure; MSP: maximum systolic pressure; POH: onset period of hypertension; RF: random forest; S/D: systolic-to-diastolic ratio; SH: serum haptoglobin; SHAP: Shapley additive explanation; UPQ: urinary protein quantification.

The data collected were used to generate 7 ML models to predict FGR developed during prenatal testing in pregnant women. The discriminative performances (including AUC, accuracy, sensitivity [recall], specificity, precision, and *F*_1_-score) of these models are shown in Table 2 and Figure 2. Among the 7 models, the RF model (AUC 0.812) had the best predictive effect for FGR, followed by the LightGBM (AUC 0.806) and GBM (AUC 0.803) models. For the RF model, the SHAP summary plots of the top 20 features (Figure 3) for the RF model are presented.

**Table 2:** The discriminative performances of these 7 models.

Model	AUC^a^ (95% CI)	Accuracy (95% CI)	Specificity (95% CI)	Sensitivity (recall; 95% CI)	Precision (95% CI)	*F*_1_-score (95% CI)
RF^b^	0.812 (0.751-0.872)	0.595 (0.449-0.76)	0.497 (0.225-0.852)	0.83 (0.511-0.997)	0.565 (0.415-0.795)	0.583 (0.441-0.787)
LGB^c^	0.806 (0.744-0.861)	0.739 (0.709-0.784)	0.756 (0.726-0.802)	0.697 (0.588-0.883)	0.541 (0.481-0.643)	0.61 (0.563-0.745)
XGB^d^	0.803 (0.727-0.862)	0.689 (0.595-0.801)	0.652 (0.471-0.874)	0.777 (0.602-0.96)	0.483 (0.467-0.997)	0.593 (0.545-0.627)
LR^e^	0.733 (0.669-0.793)	0.653 (0.513-0.716)	0.676 (0.378-0.899)	0.595 (0.24-0.857)	0.431 (0.324-0.496)	0.504 (0.472-0.581)
MLP^f^	0.701 (0.647-0.744)	0.645 (0.571-0.725)	0.662 (0.478-0.819)	0.603 (0.395-0.793)	0.421 (0.375-0.997)	0.502 (0.438-0.652)
SVM^g^	0.699 (0.643-0.794)	0.345 (0.285-0.536)	0.104 (0.0-0.468)	0.933 (0.7-1.0)	0.303 (0.231-0.426)	0.452 (0.348-0.524)
NB^h^	0.697 (0.651-0.746)	0.721 (0.706-0.737)	0.89 (0.864-0.916)	0.309 (0.208-0.397)	0.531 (0.475-0.623)	0.394 (0.298-0.473)

^a^AUC: area under the curve.

^b^RF: random forest.

^c^LGB: light gradient boosting.

^d^XGB: extreme gradient boosting.

^e^LR: logistic regression.

^f^MLP: multilayer perceptron.

^g^SVM: support vector machine.

^h^NB: naive Bayes.

### Identiﬁcation of the Final Model

**Figure 4:**
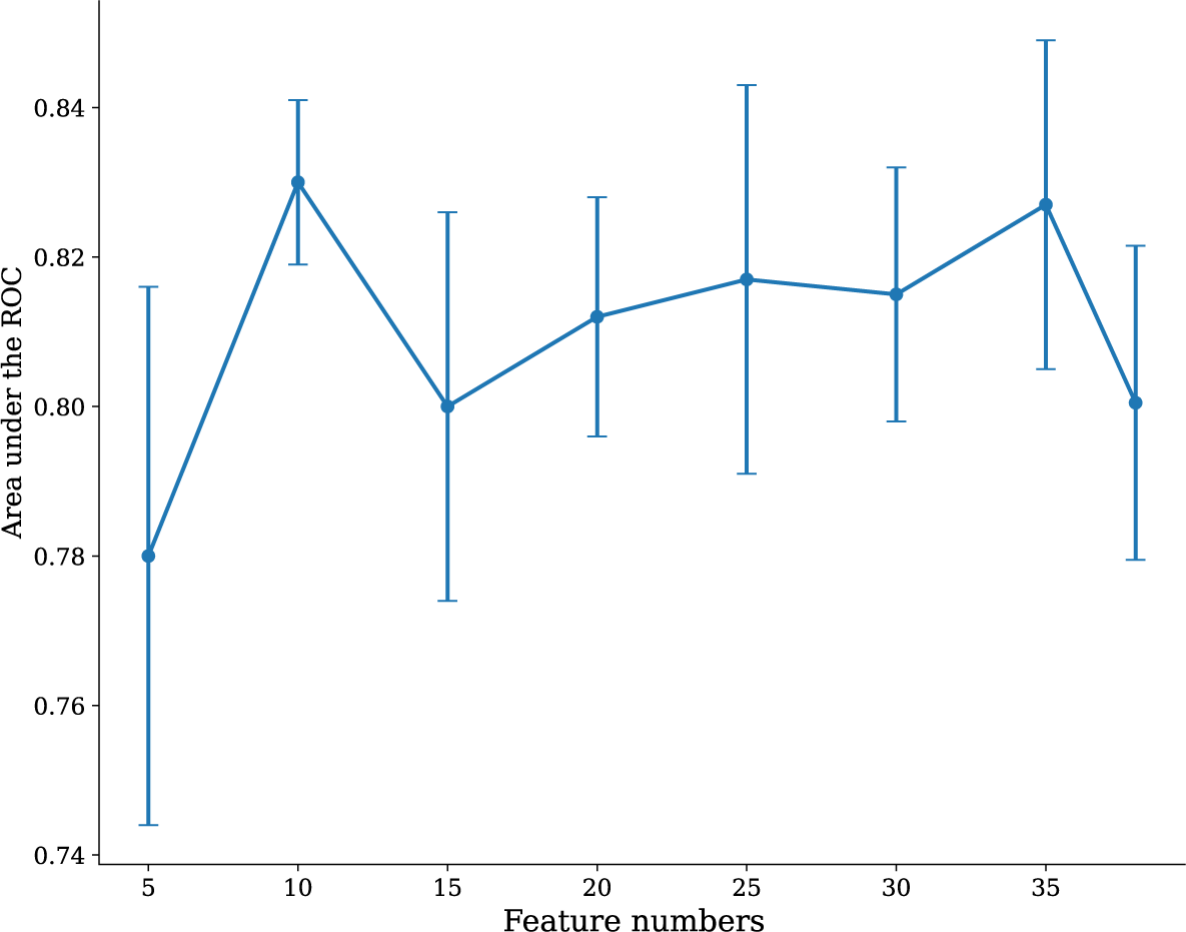
Random forest model AUC value changes with different numbers of features. AUC: area under the curve; ROC: receiver operating characteristic.

In order to study the influence of the number of features selected during model construction on model performance, we added features to the model training process step by step according to the order of feature importance shown by SHAP. The ﬁnal model was identiﬁed during the feature reduction of the RF model. As displayed in Figure 4, the 38-feature model was signiﬁcantly better than the 5-feature model (△AUC=0.036; *P*=.004), but not signiﬁcantly better than the 10-feature model (△AUC=0.011; *P*=.16) in predicting FGR in prenatal testing in a pregnant woman.

**Figure 5:**
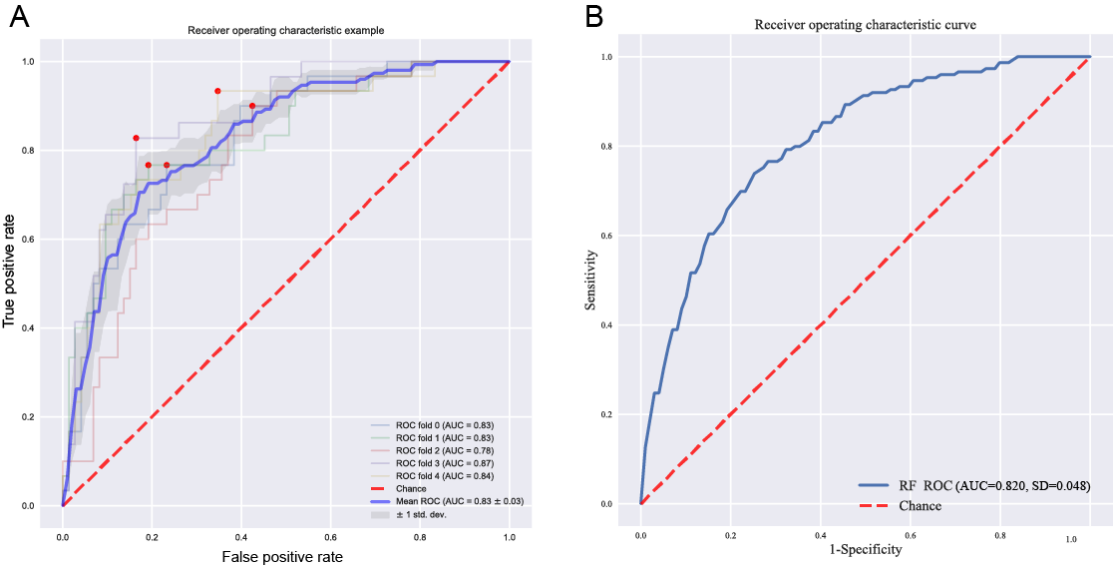
Performance of the 10-feature RF final model. (A) The 5-fold cross-validation AUC of the final model. (B) The external validation AUC. AUC: area under the curve; RF: random forest; ROC: receiver operating characteristic.

The 10-feature model had a good net beneﬁt and a high threshold probability, comparable to the 38-feature model. Hence, we focused on the 10-feature RF model. Considering the adverse effect of FGR predicted by fetal sex on sociodemographic sex ratio, we retained the other 9 features—urinary protein quantification (UPQ; mg/24 h), gestational week of delivery (GWD), umbilical artery S/D (free segment), AFI (mm), triglyceride (mmol/L), D-dimer (mg/L), weight (kg), height (cm), and maximum systolic pressure (MSP; mm Hg)—as the ﬁnal model for further analysis. For the model building process referred to 513 patients (Figure 5A), after 5-fold cross-validation, the ﬁnal RF model achieved an AUC of 0.830 with a sensitivity of 0.886, a speciﬁcity of 0.866, and an accuracy of 0.872 for predicting FGR the prenatal testing in a pregnant woman. The external validation dataset was collected and referred to 103 patients using the same standardized protocols and inclusion or exclusion criteria as the internal dataset. Statistical tests confirmed that no significant differences (all *P*>.05) existed in key variables between the 2 cohorts (Table S1 in Multimedia Appendix 1). For the external validation (Figure 5B), the ﬁnal model gave an AUC of 0.820, which was similar to that in the internal validation (ΔAUC=0.05; *P*=.48), indicating that the ﬁnal model showed great performance both in internal and external validations.

### Model Explanation

**Figure 6:**
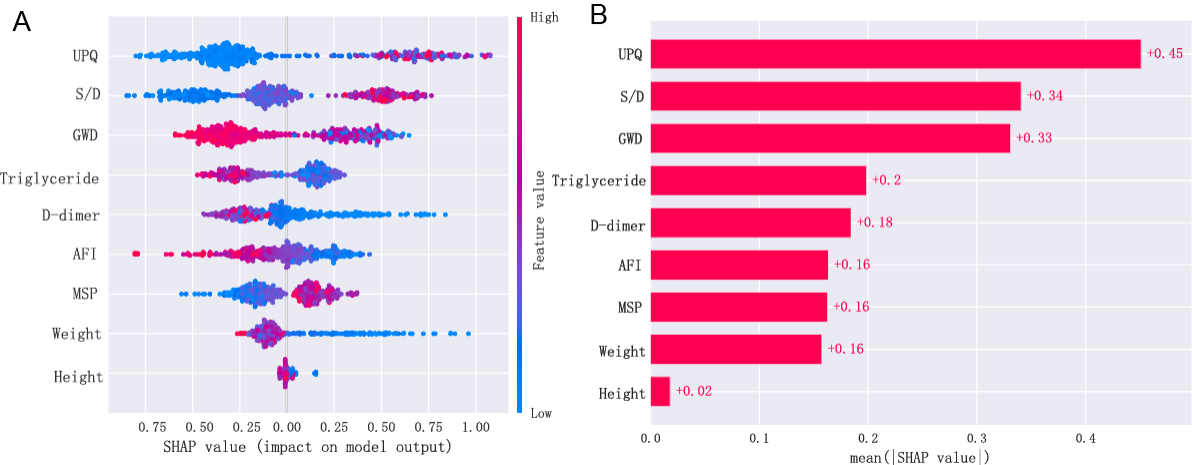
Global model explanation by the SHAP method. (A) SHAP summary dot plot. (B) SHAP summary bar plot. AFI: amniotic fluid index; GWD: gestational week of delivery; MSP: maximum systolic pressure; S/D: systolic-to-diastolic ratio; SHAP: Shapley additive explanation; UPQ: urinary protein quantification.

Since it is difﬁcult for clinicians to accept a prediction model that is not directly explainable and interpretable, the SHAP method is used to interpret the output of the ﬁnal model by calculating the contribution of each variable to the prediction. This method could provide 2 types of explanations: a global explanation of the model at the feature level and a local explanation at the individual level. The global explanation described the overall functionality of the model. As shown in SHAP summary plots (Figure 6), the contributions of the feature to the model were evaluated using the average SHAP values and exhibited in descending order. UPQ, umbilical artery S/D, and GWD showed the top three contributions (*c*=0.45, 0.34, and 0.33) to the final model.

**Figure 7:**
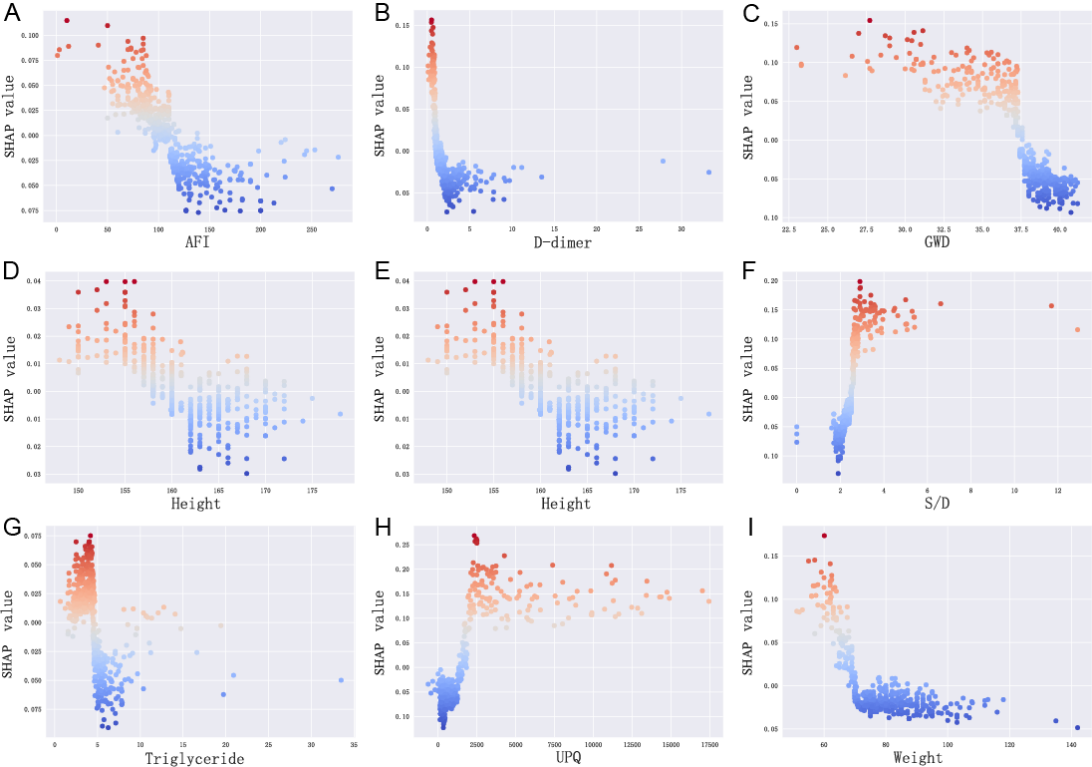
SHAP dependence plot. Each dependence plot shows how a single feature affects the output of the prediction model, and each dot represents a single patient. The darker the red color of the dot, the higher the risk of FGR, while the darker the blue color of the dot, the lower the FGR risk. AFI: amniotic fluid index; FGR: fetal growth restriction; GWD: gestational week of delivery; S/D: systolic-to-diastolic ratio; SHAP: Shapley additive explanation; UPQ: urinary protein quantification.

Additionally, the SHAP dependence plot can facilitate understanding how a single feature affects the output of the prediction model. The real values versus the SHAP values of these 9 features are shown in Figure 7, and SHAP values that are higher than zero correspond to a positive class prediction in the model, in other words, a higher risk of FGR. For instance, pregnant women with UPQ>2500 ml or MSP>150 mm Hg had SHAP values higher than 0, which pushed the decision toward the “FGR” class. In addition, GWD>37 weeks or AFI<100 mm pushed the decision toward the “non-FGR” class, as well as triglyceride (mmol/L) ≤5 or a high actual value ≥3 of umbilical artery S/D (free segment).

**Figure 8:**
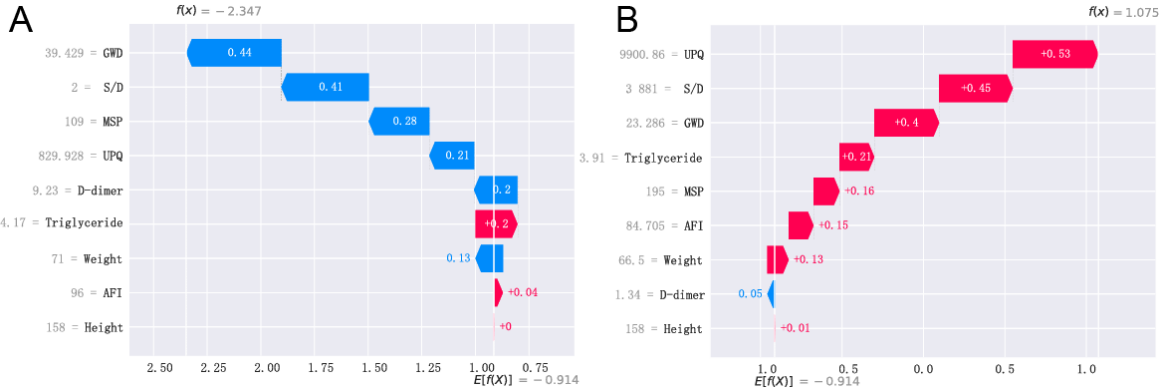
Local model explanation by the SHAP method. (A) Preeclampsia without FGR and (B) preeclampsia with FGR. AFI: amniotic fluid index; GWD: gestational week of delivery; MSP: maximum systolic pressure; S/D: systolic-to-diastolic ratio; SHAP: Shapley additive explanation; UPQ: urinary protein quantification.

Furthermore, local explanation analyzed how a certain prediction was made for a speciﬁc individual by incorporating the individualized input data. The actual measured values of features were also displayed in the waterfall plot (Figure 8). Figure 8A shows a patient whose child did not develop FGR during the pregnancy stay. As observed, the values of GWD (weeks), umbilical artery S/D (free segment), MSP (mm Hg), UPQ (mg/24 h), D-dimer (mg/L), and body weight pushed the decision toward the “non-FGR” class, but triglyceride did not. Figure 8B shows another patient whose child developed FGR during pregnancy. At present, multiple indicators such as abnormal UPQ (mg/24 h) and umbilical artery S/D (free segment) can increase the risk of FGR, pushing the decision toward the “FGR” class.

### Convenient Application for Clinical Use

The ﬁnal prediction model was implemented into the web application to facilitate its use in clinical scenarios. When the actual values of the 9 features required for the model are entered, this application will automatically predict the risk of FGR for an individual child. The web application is available in Multimedia Appendix 1.

## Discussion

### Principal Findings

Among the 7 ML models used to predict FGR from patients with preeclampsia, the RF model had the best performance with a good net beneﬁt and a high threshold probability in feature reduction. Thus, we used the RF algorithm to develop the ﬁnal model with 10 features. These features can be easily obtained or evaluated during pregnancy, so the model is promising as an early identification tool for the occurrence of FGR in pregnant women with preeclampsia.

In our study, training and testing data were collected from real clinical environments, ensuring that the model is exposed to a diverse and representative dataset. The random sampling of data helps to minimize bias and ensures that the model is robust to variations in input data. The model’s performance on real-world data validates its ability to generalize well and maintain high predictive accuracy under different input assumptions.

The ML technique has been described as a “black box” with little explanation about how predictions are derived. This may result in clinicians refusing to use it because they are hesitant to make medical decisions based on opaque information. This brought up another advantage of this study: we used the SHAP approach to explain the “black box” of ML models. The SHAP method could provide a global explanation that describes the overall functionality of a model and a local explanation that details how a certain prediction is made for an individual by inputting individualized data. Moreover, with a convenient tool based on the Streamlit framework, this prediction model can be used on a web page and shared with more clinicians.

Our ﬁnal model performed well in both internal and external validations, with an AUC of 0.83 and 0.82, respectively. Except for fetal sex, there are 9 main characteristics for predicting FGR in our model, including UPQ, MSP, triglyceride, GWD, D-dimer, weight and height of pregnant woman, umbilical artery S/D, and AFI. Urinary protein quantity and blood pressure are important indicators to reflect the severity of preeclampsia. Our prediction model found a positive correlation between urinary protein quantity, MSP, and FGR, especially when the 24-hour urinary protein quantity was greater than 2500 mg and the MSP was greater than 160 mm Hg.

A Mendelian randomization study has revealed a strong correlation between triglyceride levels in pregnant women’s blood and the occurrence of preeclampsia [27]. Previous research has found that patients with preeclampsia have higher serum triglyceride levels than normal pregnant women [28,29], and those with FGR have significantly higher levels than those without FGR [30]. Our study further discovered that when triglyceride levels in patients with preeclampsia exceed 5 mmol/L, the occurrence of FGR increases. The mechanism may be due to the presence of dyslipidemia, which may result in shallow chorionic villus implantation, ultimately leading to reduced blood flow to the placenta and uterus. This, in turn, can hinder nutrient supply and fetal development, thereby increasing the likelihood of FGR occurrence in patients with preeclampsia [14,31,32]. Bozkurt et al [33] discovered that compared with normal pregnant women, patients with preeclampsia had higher D-dimer levels, and other studies have also found a correlation between D-dimer levels and the severity of preeclampsia [34]. These findings provide strong support for our study that the risk of FGR increases when D-dimer levels in patients with preeclampsia reach 2.5 mg/L. Among ultrasound indicators of fetal growth, AFI and S/D values can reflect the occurrence of FGR. The AFI was negatively correlated with the occurrence of FGR, especially when it was less than 90 mm, and the probability of FGR is significantly increased when the S/D value is greater than 3.

Class imbalance can indeed affect the performance of ML models in multiple aspects [35]. Sampling methods such as Synthetic Minority Oversampling Technique (SMOTE) and resampling (including undersampling and oversampling) can provide solutions to this problem [17]. In this study, the number of positive (preeclampsia complicated with FGR) and negative (preeclampsia without FGR) samples are 149 and 364 in the model-building process, as well as 45 and 58 in the external dataset. The ratios between the classes are 1:2.5 and 1:1.3, respectively, which are much lower than the commonly recognized class imbalance threshold of 1:10. Therefore, we believe that the class imbalance in this study has an extremely negligible impact on the construction of the model, and thus, no special treatment is carried out.

For future FGR prediction, integrating current models based on maternal physiological, ultrasound, and genetic data into real-time clinical decision support systems is vital. These systems, connected to hospital electronic health records, can provide instant risk assessments during prenatal checkups, guiding pregnancy management. Additionally, exploring new features like pregnant women's metabolomic profiles, maternal-fetal interface microbiomes, and longitudinal placental data can enhance model accuracy. Incorporating these into existing models will likely lead to more precise FGR prediction and better maternal-fetal outcomes.

We propose integrating the predictive model into the hospital information system. During routine prenatal checkups, health care professionals can collect data through the information system, after which the model will automatically generate predictions. Health care providers can then perform interventions or follow-ups based on these results. Furthermore, using model interpretation techniques to explain the predictions will provide crucial decision-making support for clinicians. For high-risk pregnancies, doctors can develop individualized management plans in advance, such as enhanced monitoring, nutritional guidance, and medication, to improve neonatal growth and prognosis.

### Limitations

The study has several limitations. First, clinical data of patients with preeclampsia were retrospectively collected from electronic records; therefore, it is necessary to collect some prospective cases and observe the follow-up results to verify the accuracy of the model. Second, due to the lack of guidelines or consensus for selecting features for the prediction model, how many features should be included in the model remains elusive. Although more features may provide more information for the prediction model, including a large number of features may limit the clinical use of the model, and including noncausal features may reduce the accuracy of the prediction. Nevertheless, further studies are needed to simplify and validate the application of the model in clinical conditions.

### Conclusions

The prediction of FGR in patients with preeclampsia offers a valuable guide for improving perinatal outcomes. Combined with basic medical history and peripheral blood laboratory test results, this machine prediction model exhibits a reliable predictive value in predicting the occurrence of preeclampsia complicated by FGR. The model could be used as a decision-making tool to support clinicians.

## Data Availability

The datasets generated or analyzed during this study are not publicly available due to patient privacy concerns but are available from the corresponding author upon reasonable request. The use of the data is subject to relevant privacy regulations and is restricted to noncommercial research purposes. Applicants are required to provide a valid reason and usage plan for our review.

## Appendix

Multimedia Appendix 1 Parameter configurations, application for clinical use, and supporting statistical data.

## Abbreviations

AFI: amniotic fluid index
AUC: area under the curve
FGR: fetal growth restriction
GWD: gestational week of delivery
LightGBM: light gradient boosting machine
ML: machine learning
MSP: maximum systolic pressure
RF: random forest
S/D: systolic-to-diastolic ratio
SHAP: Shapley additive explanations
SMOTE: Synthetic Minority Oversampling Technique
UPQ: urinary protein quantification
XGBoost: extreme gradient boosting
