# Supplementary material for: Predictive Models Using Machine Learning to Identify Fetal Growth Restriction in Patients With Preeclampsia: Development and Evaluation Study
Source: J Med Internet Res. 2025 May 27;27:e70068. doi: 10.2196/70068 (PMC12152437; doi:10.2196/70068)
Supplement: Multimedia Appendix 1 [file jmir_v27i1e70068_app1.docx]

## Parameter Configurations

Regarding the final Random Forest model used in our study, it was configured with the following key hyperparameters: n_estimators=500, max_features=2, min_samples_split=2, max_depth=10, min_samples_leaf=4, random_state=0. Other model parameters are set by default.

## Application for Clinical Use

The ﬁnal prediction model was implemented into the web application at https://predictionmodel-for-FGR.streamlit.app.

**Table S1.** The differences of features between internal and external cohorts.

|  | Internal dataset (N=513) | External dataset  (N=103) | *P* value |
| --- | --- | --- | --- |
| Age (years), mean (SD) | 31.78 (4.20) | 31.40 (4.44) | .41 |
| Onset period of hypertension (weeks) | 31.28 (9.89) | 32.02 (10.23) | .50 |
| Gestational age at diagnosis of preeclampsia (weeks) | 34.02 (8.37) | 36.09 (4.03) | .13 |
| Gestational week of delivery (weeks) | 36.91 (3.00) | 37.12 (2.94) | .51 |
| Maximum systolic pressure(mmHg) | 154.63 (12.72) | 158.77 (77.86) | .59 |
| Maximum diastolic pressure(mmHg) | 101.51 (8.62) | 103.72 (46.73) | .63 |
| Height(cm) | 161.09 (4.09) | 161.09 (4.79) | .99 |
| Weight(kg) | 77.91 (12.43) | 77.68 (11.53) | .85 |
| Pre-pregnancy BMI(kg/m2) | 24.54 (4.01) | 24.32 (4.14) | .62 |
| Weight gain during pregnancy(kg) | 13.79 (6.05) | 14.59 (6.87) | .27 |
| Urinary protein quantification(mg/24h) | 2138.05 (3660.53) | 1857.95 (2872.86) | .39 |
| Platelets(*10^9/L) | 211.39 (59.09) | 194.01 (64.01) | .19 |
| Hematocrit(%) | 37 (3.4) | 36.17 (16.91) | .65 |
| Hemoglobin(g/L) | 120.59 (11.48) | 117.90 (15.53) | .091 |
| Total protein(g/L) | 60.51 (5.38) | 58.28 (6.40) | .088 |
| Albumin(g/L) | 33.36 (3.38) | 31.54 (4.18) | .069 |
| Globulin(g/L) | 29.57 (23.43) | 26.78 (3.87) | .25 |
| ALT(U/L) | 23.39 (62.35) | 23.41 (43.61) | >0.99 |
| AST(U/L) | 26.12 (38.09) | 32.80 (28.19) | .14 |
| ALP(U/L) | 150.58 (53.54) | 159.36 (78.71) | .27 |
| Total bilirubin (umol/L) | 11.13 (2.93) | 12.21 (13.80) | .43 |
| Uric acid (umol/L) | 387.54 (109.23) | 379.70 (103.79) | .48 |
| D-dimer (mg/L) | 1.76 (1.20) | 2.07 (2.40) | .19 |
| Fibrinogen (mg/L) | 4.87 (3.11) | 4.26 (1.07) | .48 |
| Total cholesterol (mmol/L) | 6.69 (1.15) | 6.98 (8.16) | .72 |
| Triglyceride (mmol/L) | 4.49 (1.85) | 4.69 (2.60) | .45 |
| serum haptoglobin (HP) (g/L) | 0.65 (0.38) | 0.66 (0.35) | .81 |
| Amniotic fluid Index(mm) | 111.44 (28.87) | 111.72 (35.72) | .94 |
| Umbilical artery S/D (free segment) | 2.43 (0.58) | 2.46 (0.88) | .69 |
| Chronic hypertension (hypertension diagnosed before 20 weeks of pregnancy) | 54 (11.2) | 11 (10.3) | .88 |
| History of FGR | 10 (2.0) | 1 (0.9) | .87 |
| History of cesarean section | 125 (25.9) | 23 (21.6) | .92 |
| History of adverse pregnancy | 93 (19.2) | 23 (21.6) | .94 |
| Primipara | 279 (57.8) | 42 (39.6) | .96 |
| Combined with gestational diabetes | 148 (30.7) | 32 (30.1) | .97 |
| Family history of hypertension | 79 (16.3) | 22 (20.7) | .87 |
| Fetal sex | 255 (52.9) | 60 (56.6) | .96 |

FGR: fetal growth restriction.

S/D: systolic-to-diastolic ratio.

ALT: Alanine transferase

AST: Aspartate transaminase

ALP: Alkaline phosphatase

Continuous values were presented as mean (SD) and categorical values were presented as n (%).
